# Histopathological analysis of sinonasal lesions associated with chronic rhinosinusitis and comparison with computed tomography diagnoses

**DOI:** 10.12669/pjms.36.2.1453

**Published:** 2020

**Authors:** Sultan Abdulwadoud Alshoabi, Abdulkhaleq Ayedh Binnuhaid, Moawia Bushra Gameraddin, Kamal Dahhan Alsultan

**Affiliations:** 1 Dr. Sultan Abdulwadoud Alshoabi, MBBS, MD. Department of Diagnostic Radiology Technology, College of Applied Medical Sciences, Taibah University, Almadinah Almunawwarah, Kingdom of Saudi Arabia; 2 Dr. Abdulkhaleq Ayedh Binnuhaid, MD. Department of Specialized Surgery, Radiology Section, Faculty of Medicine, Hadhramout University, Hadhramout Governorate, Republic of Yemen; 3 Dr. Moawia Bushra Gameraddin, PhD. Department of Diagnostic Radiology Technology, College of Applied Medical Sciences, Taibah University, Almadinah Almunawwarah, Kingdom of Saudi Arabia; 4 Dr. Kamal Dahhan Alsultan, PhD. Department of Diagnostic Radiology Technology, College of Applied Medical Sciences, Taibah University, Almadinah Almunawwarah, Kingdom of Saudi Arabia

**Keywords:** Chronic rhinosinusitis, Clinical diagnosis, Computed tomography, Histopathology, Nasal polyp

## Abstract

**Background &Objective:**

Chronic rhino sinusitis (CRS) is an inflammatory condition of the paranasal sinuses and the nasal passage lasting more than three months either with or without sinonasal polyps. This study aimed to report the common sinonasal lesions associated with CRS according to the histopathology results, to compare between clinical and histopathological diagnoses, and to compare between radiological and histopathological diagnoses of the sinonasal lesions.

**Methods:**

A retrospective study of the electronic records of 82 patients diagnosed with CRS with nasal polyps. All patients underwent endoscopic sinus surgery and histopathological examination of surgical biopsies. The collected data were analyzed using SPSS program. Coparison between clinical and histopathological diagnoses was done. This study was conducted at Alsafwa Consultative Medical center (ACMC) in Almukalla city, Hadhramout province in Republic of Yemen.

**Results:**

Out of 82 patients, the ages ranged from 4 to 90 years (mean: 34.48±17.74 years), and 54.88% were females. Inflammatory polyps were the most common lesion (31.4%), then allergic polyps (30.5%). Nasopharyngeal carcinoma (NPC) was reported in 9.8% of the lesions and all were unilateral. The results revealed strong compatibility between clinical and histopathological diagnoses (p<0.001, kappa= 0.215), and significant compatibility between radiological and histopathology diagnoses (p=0.007).

**Conclusion:**

Inflammatory and allergic polyps are the most common benign bilateral lesions associated with chronic rhinosinusitis, which can be correctly diagnosed clinically in most cases. Unilateral nasal polyps have high rates of malignancies and should be check carefully by endoscopy and histopathology. Computed tomography has some pitfalls in diagnosing of fungal sinusitis.

## INTRODUCTION

Chronic rhino sinusitis (CRS) is an inflammatory condition of the paranasal sinuses and the nasal passage lasting more than three months with two or more of the following manifestations; nasal discharge, swelling nasal mucosa, pain and impaired smell.^1^,^2^ It is a common disease that affects more than 10% of peoples in Europe.^3^

CRS can be divided into two phenotypes, either inflammation associated with polyps (CRSwNP) or without polyps (CRSsNP).^1^,^2^,^4^ In western countries, about 80% of CRS are CRSsNP and only 20% CRSwNP.^5^ Based on eosinophilic inflammation: CRSwNP can be subdivided into eosinophilic (Eos CRSwNP) and non-eosinophilic (Non-Eos CRSwNP).^4^,^6^ Nasal polyps are tear-drop like growths that form in the nasal cavity or in the paranasal sinuses (PNS). They often accompanied allergies and chronic infections.^7^

Diagnosis and treatment of the airway diseases becomes increasingly important as different phenotypes described.^6^,^8^ CRS and asthma are characterized by recurrent symptoms leading to frequent treatment and high prescription costs. About 50% of patients with CRS have asthma and 80% of asthmatic patients suffering from CRS.^9^ Their medical importance arises from impaired sleep quality and depression symptoms accompanying them.^10^,^11^

CRS with or without NPs can be diagnosed using nasal endoscopy.^3^ The Europian guidelines on rhinosinusitis and nasal polyps (EPOS 2012) determined computed tomography (CT) as the primary imaging modality to assess the intensity of the inflammatory lesions of the nose and PNS. Bone window is the most suitable to avoid missing lesions in the PNS.^12^

The aim of this study were, firstly, to report the common causes of sinonasal lesions in suspected patients of CRSwNP according to the histopathology results, secondly, to compare between clinical and histopathological diagnoses, and tertiary, to compare between radiological and histopathological diagnoses of the sinonasal lesions. This study will be important for otorhinolarynologists to suggest the cause and select the best management for these patients.

## METHODS

### Patients Selection

This cross-sectional retrospective study was conducted at Alsafwa Consultative Medical center (ACMC) in Almukalla city, Hadhramout province in Republic Yemen. Data of 82 patients with CRS who underwent histopathological diagnoses from June 2016 to June 2019 were retrieved from the computerized database of the Radiology and Medical Imaging Department.

**Fig.1:**
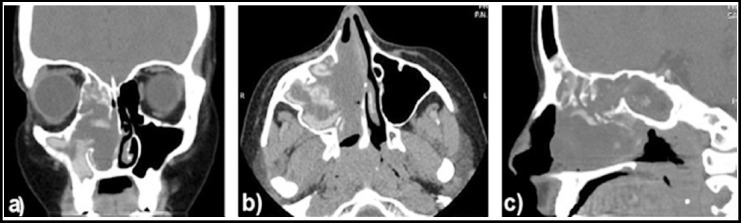
Computed tomography images of adult female patient revealed multiple polyps in the right paranasal sinuses a) coronal section, b) axial section, and c) sagittal section.

**Fig.2:**
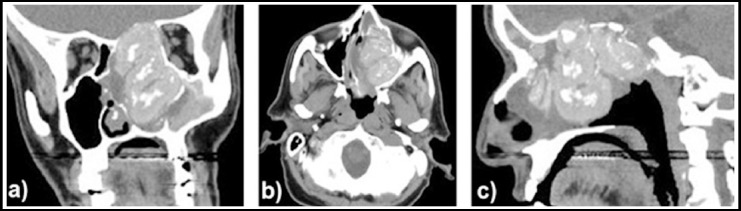
Computed tomography images of adult male patient revealed multiple polyps in the left paranasal sinuses a) coronal section, b) axial section, and c) sagittal section.

### Variables Assessed

Electronic medical records were reviewed and clinical and histopathologic diagnoses were collected. The patients were clinically diagnosed with CRSwNP by highly-qualified otorhinolaryngologist. All the patients underwent histopathology analysis of biopsies by highly-qualified pathologist with 20 years’ post-doctorate experience. Only 30 patients underwent multislice non-enhanced computed tomography (NECT) of the paranasal sinuses (PNS). Radiological diagnoses were done by board-qualified radiologist with 10 years’ post-doctorate experience. The clinical and histopathological diagnoses were compared. After deleting the cases with no available NECT, the radiological and histopathological diagnoses of remained 30 patients were compared. Inclusion criteria include all patients clinically diagnosed as CRSwNP and underwent endoscopic sinus surgery and biopsies were taken. Exclusion criteria include all patients clinically diagnosed as CRSsNP without any interventional procedures.

### Ethics

This study was approved by the institutional ethics committee at Alsafwa Consultative Medical center (ACMC) in Almukalla under the protocol No. 30-7-100/1. Confidentiality of the patients was assured.

### Statistical Analysis

Data are presented as frequency and percentage for continuous variables and mean ± SD for descriptive variables. Cross-tabulation using the chi-square test and measurement of agreement using the Kappa test were performed to compare the clinical and histopathological diagnoses. Data analysis was performed using SPSS version 23 (Armonk, NY: IBM Corp, 2015). P-value was assumed to be significant when < 0.05.

## RESULTS

Among 82 patients with CRS involved in this study, the ages ranged from 4 to 90 years (mean: 34.48±17.74 years), and 45 (54.88%) were females. Most of the patients were in the second to the fifth decades and peaking at the third decade (p<0.001) (Table-I). The most common lesion was inflammatory polyps (31.4%), and allergic polyps (30.5%). Nasopharyngeal carcinoma (NPC) was 9.8% of the lesions (Table-II).

**Table-I:** Sociodemographic distribution of the patients.

Variable	Number	Percentage	Significance
Female	45	54.88%	p=0.377
Male	37	45.12%	
First decade	6	7.3%	p<0.001
Second decade	11	13.4%	
Third decade	25	30.5%	
Fourth decade	13	15.9%	
Fifth decade	14	17.1%	
Sixth decade	8	9.8%	
Seventh decade	2	2.4%	
Eighth decade	3	3.7%	

**Table-II:** Histopathologic results of the lesions associated with CRS.

Diagnosis	Number	Percentage
Chronic sinusitis only	12	14.6
Inflammatory polyp	26	31.7
Allergic polyp	25	30.5
Antrochoanal polyp	3	3.7
NPC	8	9.8
Others	8	9.8

Total	82	100.0

The results revealed strong compatibility between the clinical diagnoses and the histopathology results, (p<0.001) and the measure of agreement kappa= 0.215 (Table-III). The results also revealed significant compatibility between CT diagnoses of the radiologist and the histopathology results (p<0.001) (Table-IV).

**Table-III:** Cross tabulation between clinical and histopathologic diagnoses.

Clinical diagnoses	Histopathological diagnoses						

	Chronic sinusitis	Inflammatory polyp	Allergic polyp	Antrochoanal polyp	NPC	Others	Total

	No. (%)	No. (%)	No. (%)	No. (%)	No. (%)	No. (%)	No. (%)
CRS	9 (22.5)	16 (40)	12 (30)	0 (0.0)	2 (5)	1 (2.5)	40 (100)
Polyposis	0 (0.0)	7 (58.3)	4 (33.3)	0 (0.0)	0 (0.0)	1 (8.3)	12 (100)
Antrochoanal polyp	1 (12.5)	2 (25)	2 (25)	3 (37.5)	0 (0.0)	0 (0.0)	8 (100)
NPC	2 (22.2)	0 (0.0)	0 (0.0)	0 (0.0)	6 (66.7)	1 (1.1)	9 (100)
Others	0 (0.0)	1 (7.7)	5 (53.8)	0 (0.0)	0 (0.0)	5 (38.5)	13 (100)
Total	12 (14.6)	26 (31.7)	25 (30.5)	3 (3.7)	8 (9.8)	8 (9.8)	82 (100)

Table revealed strong compatibility between clinical and histopathological diagnoses. (p<0.001, kappa= 0.215).

**Table-IV:** Cross tabulation between Radiological and histopathologic diagnoses.

CT diagnoses	Histopathological diagnoses					

	Chronic sinusitis	Inflammatory polyp	Allergic polyp	NPC	Others	Total

	No. (%)	No. (%)	No. (%)	No. (%)	No. (%)	No. (%)
Nasal polyp	0 (0.0)	1 (50)	1 (50)	0 (0.0)	0 (0.0)	2 (100)
Fungal sinusitis	0 (0.0)	1 (33.3)	0 (0.0)	0 (0.0)	2 (66.7)	3 (100)
Sinonasal polyposis	1 (6.3)	5 (31.3)	9 (56.3)	0 (0.0)	1 (6.3)	16 (100)
Antrochoanal polyp	2 (50)	2 (50)	0 (0.0)	0 (0.0)	0 (0.0)	4 (100)
Carcinoma	2 (100)	0 (0.0)	0 (0.0)	0 (0.0)	0 (0.0)	2 (100)
Others	1 (33.3)	1 (33.3)	0 (0.0)	1 (33.3)	0 (0.0)	3 (100)
Total	6 (20)	10 (33.3)	10 (33.3)	1 (3.3)	3 (10)	30 (100)

Table revealed significant compatibility between radiological and histopathological diagnoses (p=0.007).

## DISCUSSION

Chronic rhinosinusitis is a widespread health problem worldwide. It is associated with sinonasal polyps in 20% of cases. This study reported strong association between benignity and bilateral sinonasal lesions and the vice versa with malignant lesions. The results revealed strong compatibility between clinical and histopathological diagnoses of the sinonasal lesions.

The present results demonstrate that inflammatory and allergic polyps were the most common lesions associated with CRS. This result is consistent with Stevens et al., who reported that CRSwNP is frequently associated with allergic rhinitis and asthma. The association between CRSwNP and asthma has been extensively defined. Asthma was reported in 26-48% of patients with CRSwNP while CRSwNP was estimated to occur only in 7% of asthmatic patients.^13^

In the current study, most of the sinonasal lesions were benign. This result is compatible with Dutta et al, who reported that polypoid sinonasal masses are mostly benign.^14^ In our results, inflammatory polyp was the most common lesion and hemangioma was the most common benign neoplasm. These results were compatible with the results of Singh et al., who reported the same findings.^15^

Our results reported eight cases of nasopharyngeal carcinoma those were unilateral and presented by unilateral nasopharyngeal manifestations. This result is compatible with Wong et al., who reported that unilateral nasal polyps have higher rates of malignancies than bilateral lesions.^16^ This result also consistent with the results of Eckhoff et al., who reported significant association between unilateral sinonasl lesions and diagnoses of benign and malignant neoplasms.^17^ The result also consistent with Arslan et al., who reported that any unilateral nasal mass should be examined by histopathology to exclude neoplasms.^18^ Belli et al, also recommended pathological examination for all unilateral sinonasal lesions at any age.^19^

In this study, the results revealed strong compatibility between clinical and radiological diagnoses for bilateral sinonasal lesions. All cases diagnosed as sinonasal polyposis were inflammatory and allergic but no one was malignant. This result is consistent with Wong et al., who reported that discrepancies between clinical and histopathological diagnoses of bilateral nasal polyps is very low.^16^

In our results, we reported significant accuracy for CT to diagnose sinonasal lesions with two pitfalls in diagnosing fungal sinusitis. This result is consistent with Kandukuri et al., who reported CT as the modality of choice for evaluating inflammatory, benign and malignant sinonasal lesions with potential pitfalls in differentiating fungal sinusitis from dense secretions.^20^

Other pitfalls of CT were mentioned by Popolizio et al., who reported that Foreign body (FB) in the sinus with matted fungal hyphae can appear as a mass on CT and also thickened inflamed sinus mucosa may enhance after contrast administration.^21^

### Limitations of this study

This study is limited by its retrospective nature, with no available computed tomography in all cases. It was performed in a single center.

## CONCLUSION

Inflammatory and allergic polyps are the most common benign bilateral lesions of the sinonasal area, which can be correctly diagnosed clinically in most cases. Further confirmation by histopathology should be reserved for doubtful cases only. Unilateral nasal polyps have high rates of malignancies and should be check carefully by endoscopy and histopathology. Computed tomography has some pitfalls in diagnosing of fungal sinusitis.

### Authors Contribution:

**SAA:** Conceived and designed the study; organised, and analysed data; and wrote the initial and final drafts of the manuscript.

**AAB:** Working computed tomography reports and collected data.

**MBG:** Participated in data analysis.

**KDA:** Review the final manuscript. All authors have approved the final draft and are responsible for the content of the manuscript.

### Abbreviations:

**CRS:** Chronic rhino sinusitis, **CRSwNP:** CRS with nasal polyps, **CRSsNP:** CRS without nasal polyp, **Eos CRSwNP:** CRSwNP and eosinophilic inflammation, **PNS:** paranasal sinuses,

**EPOS 2012:** European position paper on rhinosinusitis and nasal polyps 2012,

**ACMC:** Alsafwa Consultative Medical center, **NECT:** non-enhanced computed tomography, **SPSS:** Statistical Package for the Social sciences, **IBM:** International Business Machines,

**NY:** New York, **NPC:** Nasopharyngeal carcinoma.
